# Klebsiella Species and Enterobacter cloacae Isolates Harboring *bla*_OXA-181_ and *bla*_OXA-48_: Resistome, Fitness Cost, and Plasmid Stability

**DOI:** 10.1128/spectrum.03320-22

**Published:** 2022-12-01

**Authors:** Samiratu Mahazu, Isaac Prah, Yusuke Ota, Takaya Hayashi, Yoko Nukui, Masato Suzuki, Yoshihiko Hoshino, Yukihiro Akeda, Toshihiko Suzuki, Tomoko Ishino, Anthony Ablordey, Ryoichi Saito

**Affiliations:** a Department of Molecular Microbiology, Tokyo Medical and Dental University, Tokyo, Japan; b Department of Parasitology and Tropical Medicine, Tokyo Medical and Dental University, Tokyo, Japan; c Department of Molecular Virology, Tokyo Medical and Dental University, Tokyo, Japan; d Department of Infection Control and Prevention, Tokyo Medical and Dental University Hospital, Tokyo, Japan; e Department of Infection Control and Laboratory Medicine, Kyoto Prefectural University of Medicine, Kyoto, Japan; f Antimicrobial Resistance Research Center, National Institute of Infectious Diseases, Tokyo, Japan; g Department of Mycobacteriology, Leprosy Research Center, National Institute of Infectious Diseases, Tokyo, Japan; h Department of Bacteriology I, National Institute of Infectious Diseases, Tokyo, Japan; i Department of Bacterial Pathogenesis, Tokyo Medical and Dental University, Tokyo, Japan; j Department of Bacteriology, Noguchi Memorial Institute for Medical Research, University of Ghana, Accra, Ghana; University of Pretoria

**Keywords:** oxacillinase-48 (OXA-48), oxacillinase-181 (OXA-181), fitness cost, plasmid stability, IncX3, IncL

## Abstract

IncX3 and IncL plasmids have been named as catalysts advancing dissemination of *bla*_OXA-181_ and *bla*_OXA-48_ genes. However, their impact on the performance of host cells is vastly understudied. Genetic characteristics of *bla*_OXA-48_- and *bla*_OXA-181_-containing Klebsiella pneumoniae (EFN299), Klebsiella quasipneumoniae (EFN262), and Enterobacter cloacae (EFN743) isolated from clinical samples in a Ghanaian hospital were investigated by whole-genome sequencing. Transfer of plasmids by conjugation and electroporation, plasmid stability, fitness cost, and genetic context of *bla*_OXA-48_, *bla*_OXA-181_, and *bla*_DHA-1_ were assessed. *bla*_OXA-181_ was carried on two IncX3 plasmids, an intact 51.5-kb IncX3 plasmid (p262-OXA-181) and a 45.3-kb IncX3 plasmid (p743-OXA-181) without replication protein sequence. The fluoroquinolone-resistant gene *qnrS1* region was also excised, and unlike in p262-OXA-181, the *bla*_OXA-181_ drug-resistant region was not found on a composite transposon. *bla*_OXA-48_ was carried on a 74.6-kb conjugative IncL plasmid with unknown ~10.9-kb sequence insertion. This IncL plasmid proved to be highly transferable, with a conjugation efficiency of 1.8 × 10^−2^. *bla*_DHA-1_ was present on an untypeable 22.2 kb genetic structure. Plasmid stability test revealed plasmid loss rate between 4.3% and 12.4%. The results also demonstrated that carriage of IncX3-*bla*_OXA-181_ or IncL-*bla*_OXA-48_ plasmids was not associated with any fitness defect, but rather an enhanced competitive ability of host cells. This study underscores the significant contribution of IncX3 and IncL plasmids in the dissemination of resistance genes and their efficient transfer calls for regular monitoring to control the expansion of resistant strains.

**IMPORTANCE** The growing rate of antibiotic resistance is an important global health threat. This threat is exacerbated by the lack of safe and potent alternatives to carbapenems in addition to the slow developmental process of newer and effective antibiotics. Infections by carbapenem-resistant Gram-negative bacteria are becoming almost untreatable, leading to poor clinical outcomes and high mortality rates. OXA-48-like carbapenemases are one of the most widespread carbapenemases accounting for resistance among Enterobacteriaecae. We characterized OXA-48- and OXA-181-producing Enterobacteriaecae to gain insights into the genetic basis and mechanism of resistance to carbapenems. Findings from the study showed that the genes encoding these enzymes were carried on highly transmissible plasmids, one of which had sequences absent in other similar plasmids. This implies that mobile genetic elements are important players in the dissemination of resistance genes. Further characterization of this plasmid is warranted to determine the role of this sequence in the spread of resistance genes.

## INTRODUCTION

Antibiotic resistance is a global health concern requiring concerted efforts for its control (1, 2). Gram-negative bacteria have become resistant to a wide spectrum of antibiotics mainly as a consequence of structural adaptations and production of enzymes, such as extended spectrum beta lactamases (ESBLs) and carbapenemases, that inactivate antibiotics (3). Particularly alarming is the growing incidence of carbapenem-resistant Enterobacteriaceae infections associated with limited treatment options (4).

Carbapenems are one of the safest and most effective antibiotics, with a broad range of antibacterial activity, and were used as monotherapy, solely to treat severe infections. The emergence and global dissemination of ESBL-producing bacteria necessitated increased clinical use of carbapenems for empirical treatment, which consequently resulted in the emergence of carbapenem-resistant strains (5). A recent meta-analysis attributed 26% to 44% mortality rate to infections with carbapenem-resistant strains and found K. pneumoniae as the main causative organism of those lethal infections (6).

Among the carbapenemases described so far, OXA-48, one of the most widespread, is highly endemic in the Middle East, North Africa, and Europe and usually found in Escherichia coli and K. pneumoniae (7, 8). The pervasiveness and rapid spread of OXA-48 has been ascribed in part to reduced fitness burden exerted upon host cells coupled with stable maintenance of the plasmids, and to efficient horizontal gene transfer (7). At present, about 39 OXA-48 variants have been described (https://www.ncbi.nlm.nih.gov/pathogens/refgene/#oxa-48), and notwithstanding that they vary by only a few amino acid substitutions, these enzymes exhibit profound diversity in their β-lactam hydrolysis, genetic context, and horizontal gene transfer (HGT) (8). OXA-181 has become the second most common among the variants after OXA-48 and has been reported in several other parts of the world as a significant cause of hospital-acquired infections, after its first description in 2007 in India (9, 10).

Carbapenemases have different epidemiological importance depending on their origin and spread (5), and although a modest number of studies have reported on the clinical occurrence of carbapenem-resistant bacteria in Ghana, studies characterizing the mechanisms of resistance and their impact on the dissemination of resistant strains using high molecular techniques such as next-generation sequencing (NGS) are few (11–14). Availability of such epidemiological data is essential for the control of the spread of resistant strains. To this end, we studied 29 Gram-negative bacteria isolates from Ghana. Carbapenem-resistant isolates were investigated using whole-genome sequencing (WGS). Plasmid transfer and stability in bacteria as well as genetic context were evaluated.

## RESULTS

### Identification of OXA-48- and OXA-181-producing isolates.

Due to difficulty with identification of *bla*_OXA-48_-like containing strains (10), mCIM test was conducted for all isolates irrespective of the MIC of imipenem and meropenem. One *K. quasipneumoniae* isolate, EFN 262 (imipenem MIC 1 μg/mL), K. pneumoniae EFN299 (imipenem MIC 2 μg/mL), and E. cloacae EFN743 (imipenem and meropenem MIC 1 μg/mL) (Data Set S1) had a zone of inhibition diameter of 6 mm from the mCIM test, which according to the CLSI, indicates a carbapenemase producer. Additionally, EFN299 was resistant to piperacillin, cefazolin, cefotaxime, ceftazidime, cefepime, cefpodoxime, sulbactam/ampicillin, aztreonam, gentamicin, minocycline, sulfamethoxazole-trimethoprim, and levofloxacin. EFN262 and EFN743 were also resistant to six and 11 other antibiotics, respectively (Data Set S1). PCR screening detected *bla*_OXA-181_ in EFN262 and EFN743, and *bla*_OXA-48_ in EFN299. The overall rates of resistance to the various antibiotics are shown in Table 1.

**TABLE 1:** MIC 50 (MIC_50_) and MIC 90 (MIC_90_) values and antibiotics resistance percentage values of twenty-nine Gram-negative bacteria isolates

Antimicrobial agents	Breakpoint for resistance(μg/mL)	% resistance	MIC (μg/mL)		
			Range	MIC_50_	MIC_90_
Piperacillin	≥128	70.0	≤0.5 to >64	>64	>64
Cefazolin	≥8	70.0	1 to >16	>16	>16
Cefotaxime	≥4	79.3	≤0.5 to >32	32	>32
Ceftazidime	≥16	51.7	≤0.5 to >16	16	>16
Cefepime	≥16	34.5	≤0.5 to >16	8	>16
Sulbactam/Ampicillin	≥16/32	62.1	≤2/4 to >8/16	>8/16	>8/16
Cefpodoxime	≥8	82.8	≤1 to >4	>4	>4
Aztreonam	≥16	70.0	≤0.5 to >16	>16	>16
Imipenem	≥4	3.4	≤0.25 to >8	≤0.25	>8
Meropenem	≥4	10.3	≤0.25 to >8	≤0.25	2
Gentamicin	≥16	34.5	≤0.25 to >8	0.5	>8
Amikacin	≥64	0.0	≤1 to 16	2	8
Minocycline	≥16	41.4	≤0.25 to >8	8	>8
Fosfomycin	≥256	6.9	≤32 to >128	≤32	128
Sulfamethoxazole/Trimethoprim	≥76/4	79.3	≤9.5/0.5 to >38/2	>38/2	>38/2
Levofloxacin	≥2	62.1	≤0.25 to >4	>4	>4

### WGS and phylogenetic analysis.

**FIG 1:**
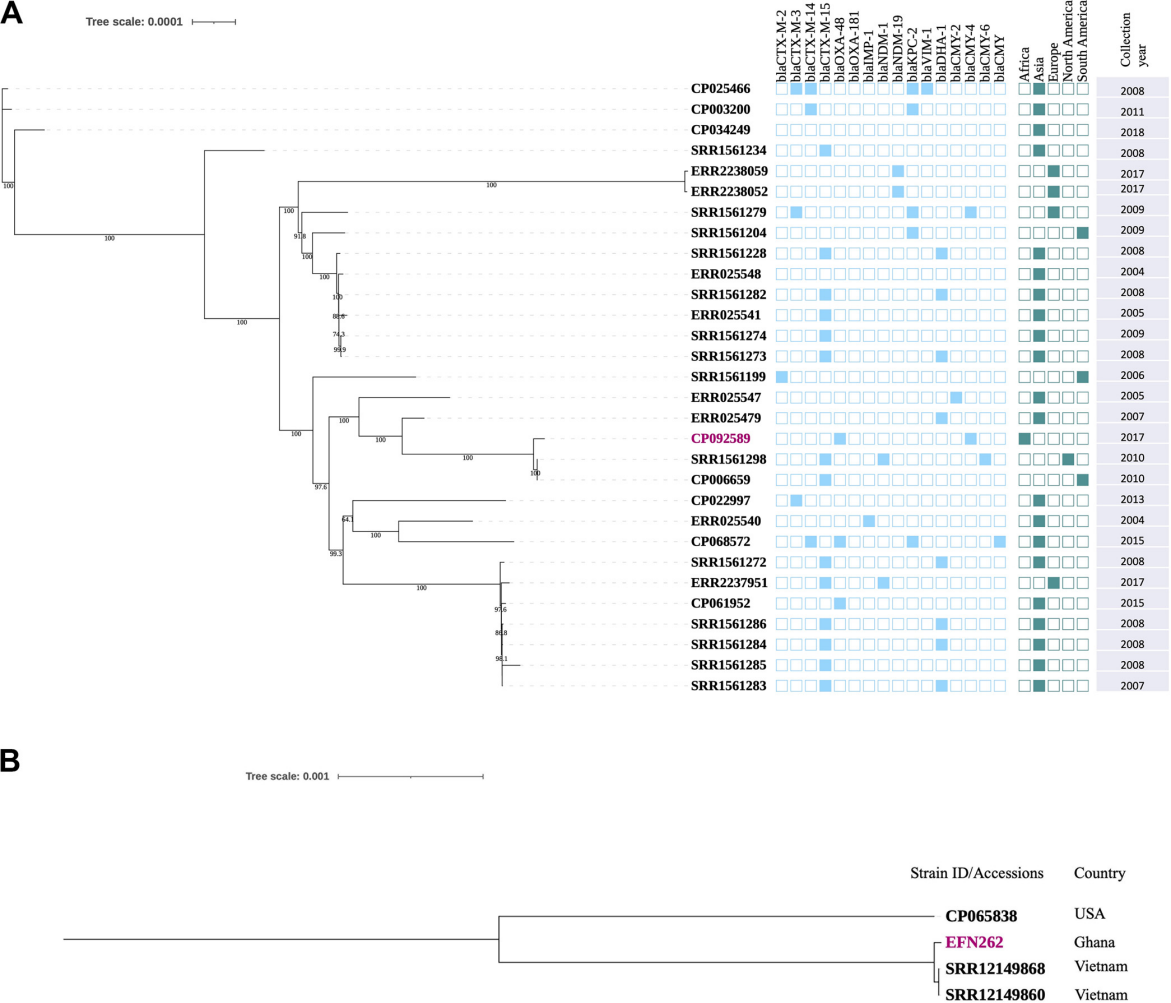
(A) Core-genome-based maximum likelihood phylogeny of 29 Klebsiella pneumoniae ST11 strains retrieved from a database Klebsiella pneumoniae database (https://bigsdb.pasteur.fr/klebsiella/), and one from Ghana (this study, highlighted in pink). Branch patterns were assessed by 1,000 bootstrap replicates. Continent of origin and antibiotic resistance genes are displayed in a binary fashion in shaded rectangles. (B) Core-genome-based maximum-likelihood phylogeny of two Klebsiella quasipneumoniae ST5015 strains from Vietnam and one from this study (Ghana). The Ghanaian strain is highlighted in pink. The strain with accession number CP065838 was added to aid construction of the tree.

Hybrid assembly generated seven contigs for EFN262, comprising a circular chromosome of length 5,283,317 bp and six circular plasmids of sizes ranging between 203,351 bp and 4,655 bp. There were eight and four contigs for EFN299 and EFN743, respectively. The PlasmidFinder database revealed that EFN262 and EN743 harbored, among others, the IncX3 plasmid replicon and EFN299, the IncL replicon. MLST assigned EFN299 to the notorious ST11 and EFN743 to ST456. EFN262 was determined to belong to an emerging ST5015. In investigating the phylogenetic relationship between ST11 K. pneumoniae from this study and others, we retrieved genomes of isolates from four continents (Asia, Europe, South America, and North America) and constructed a maximum-likelihood phylogeny. The result of the clustering suggested that strain EFN299 (CP092589) is closely related to strains from North America and South America (Fig. 1A). To determine whether the same clone of *K. quasipneumoniae* ST5015 is causing infections in Vietnam and Ghana, the two genomes (accession nos. SRR12149860 and SRR12149868) of ST5015 strains from a Vietnamese study (15) were retrieved and single nucleotide polymorphisms (SNPs) were compared between each of those two and the one genome (CP092512) from this study. The results showed the genomes differed by a high number of SNPs, 1,872 (SRR12149860 to CP092512) and 1,882 (SRR12149868 to CP092512). Roary was used to estimate the number of genes shared between the genomes. The total number of genes found in the three genomes was 5,870, and of this number, 4,744 were core genes. In spite of the large number of core genes, the phylogeny clustered the genome of the strains from Vietnam on a different branch from our strain, but also showed a close phylogenetic relationship among them indicating they may have a similar ancestry (Fig. 1B).

### Genetic environment of *bla*_OXA-181_, *bla*_OXA-48_, *bla*_DHA-1_, and plasmid phylogeny.

**FIG 2:**
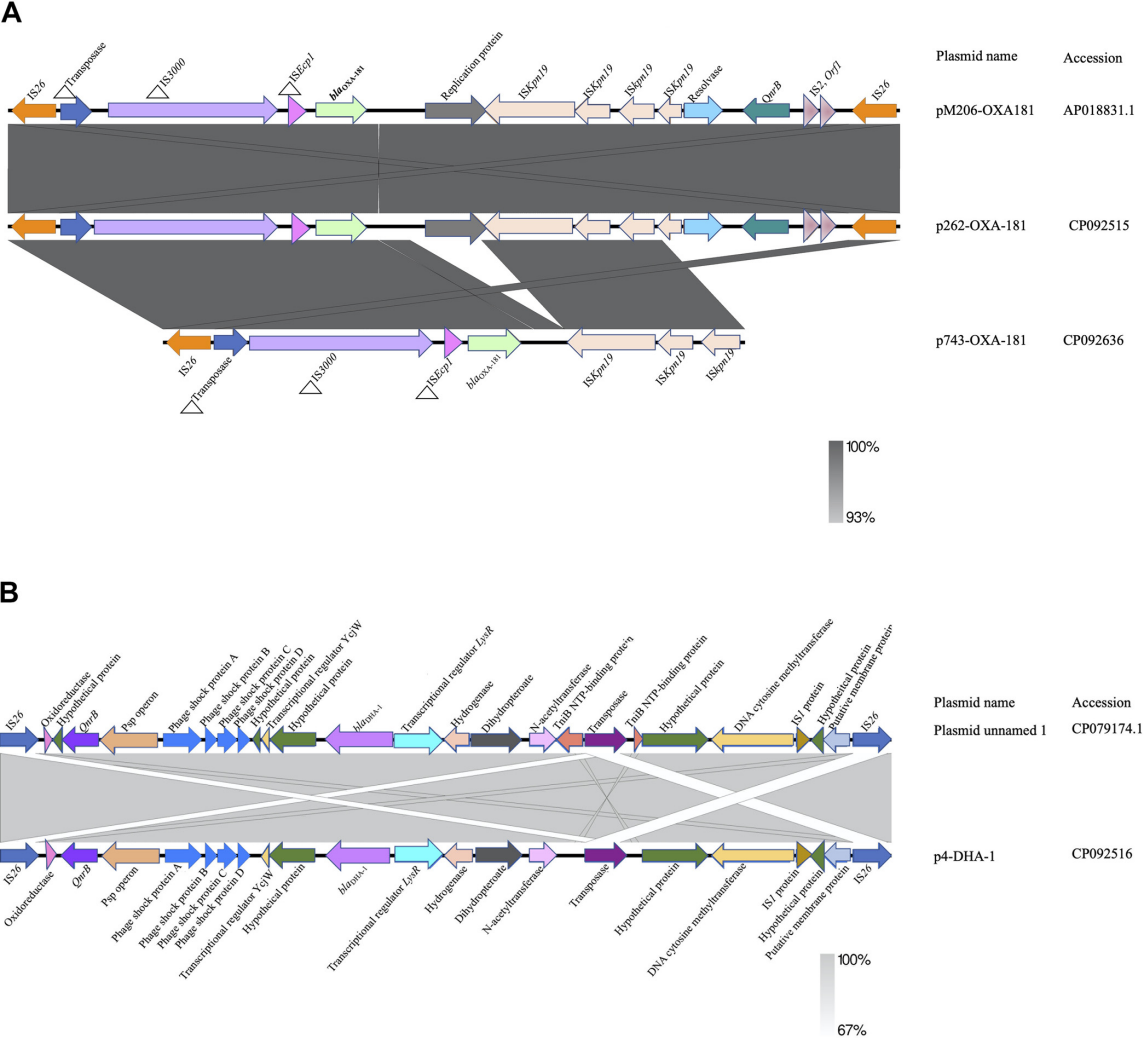
(A) Linear comparison of the genetic environment of *bla*_OXA-181_ generated by Easyfigv2.2.2. Sequences upstream and downstream of *bla*_OXA-181_ are illustrated with arrows, with arrowheads indicating the direction of transcription. The strand of p743-OXA-181 is reversed for easy comparison. Truncated structures are preceded by triangles. Different gene features are shown by the different colors. (B) Genetic context of *bla*_DHA-1_ identified in strain EFN262. Sequences upstream and downstream of *bla*_DHA-1_ are illustrated with arrows, with arrowheads indicating the direction of transcription.

**FIG 3:**
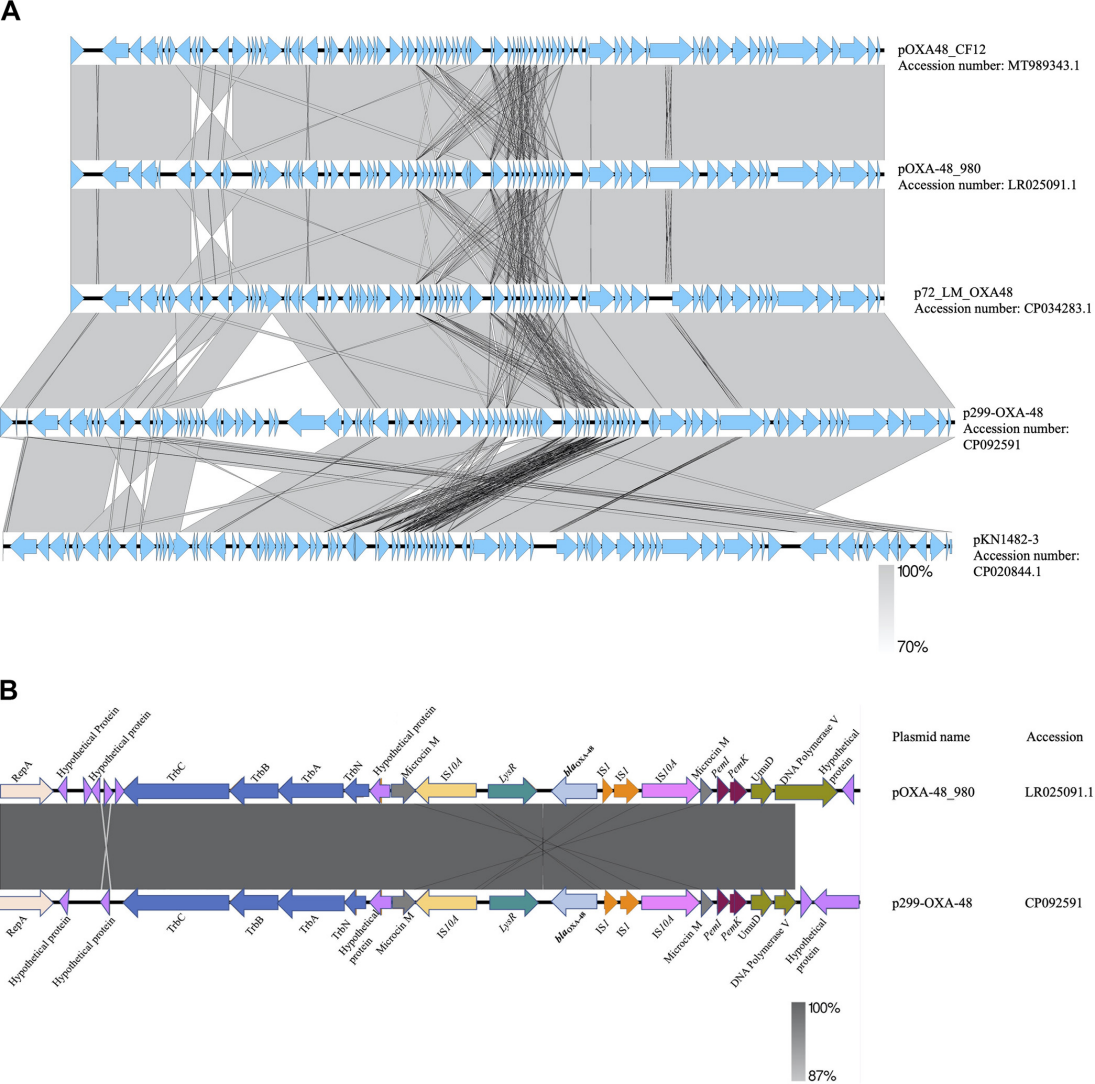
(A) Linear comparison of the entire length of IncL plasmid structures generated with Easyfigv2.2.2. (B) Linear representation of the genetic context of *bla*_OXA-48_. Genetic structures upstream and downstream are indicated as arrows. Arrowheads represent the direction of transcription. Truncated structures are preceded by triangles.

**FIG 4:**
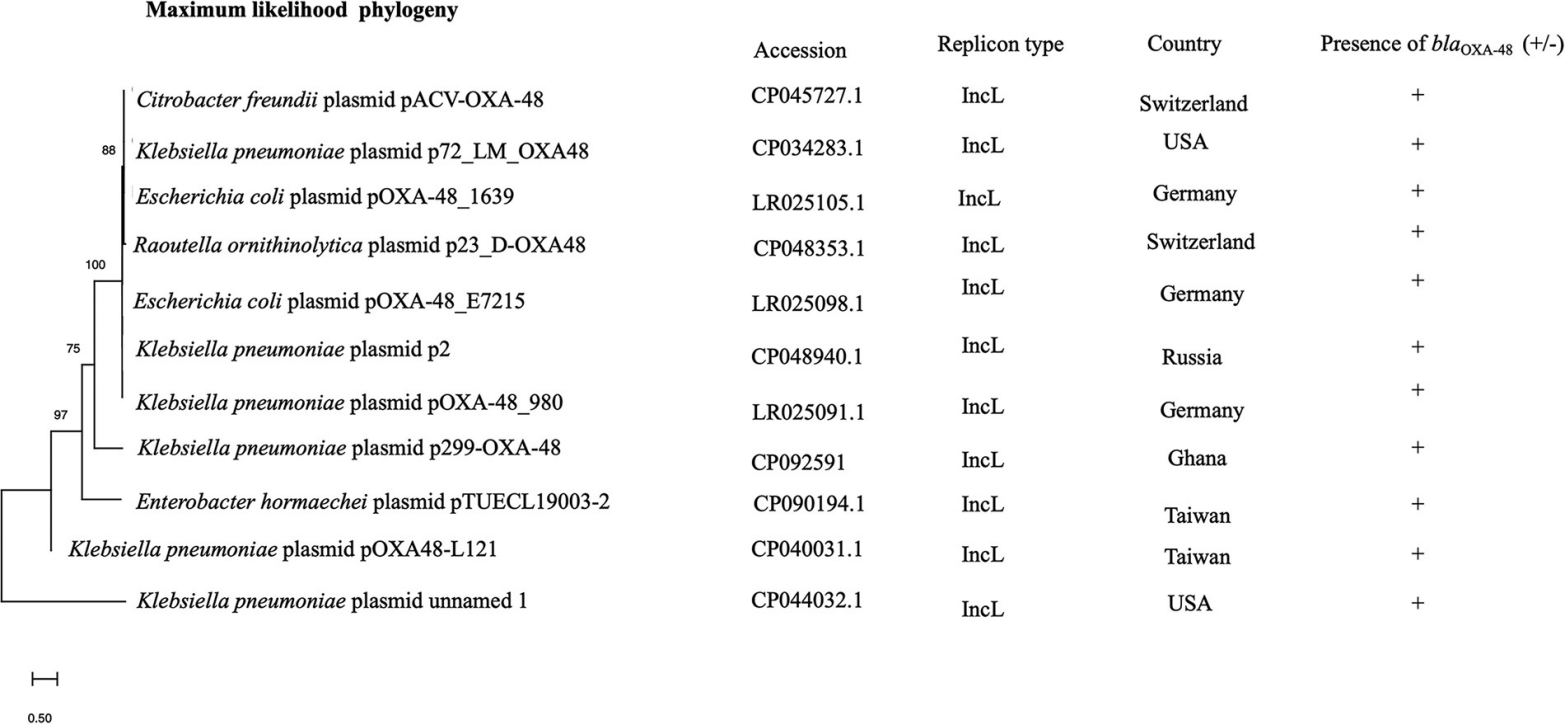
Maximum likelihood phylogeny of 10 IncL plasmids downloaded from the NCBI database and one IncL plasmid (p299-OXA-48) from this study.

*bla*_OXA-181_ was present on a 51,479-bp IncX3 plasmid, p262-OXA-181 (accession no. CP092515) and contained in the *K. quasipneumoniae* strain, but was present on a 45,279-bp IncX3 plasmid, p743-OXA-181 (accession no. CP092636) in the E. cloacae strain. The blast search tool was queried for plasmid sequences similar to sequences of the *bla*_OXA-181_-containing region in p262-OXA-181 and p743-OXA-181. The search produced a 100% query cover and sequence identity to p262-OXA-181 and p743-OXA-181. The linear plasmid sequences of p262-OXA-181 was compared to sequences of a plasmid recovered from the Enterobacter hormaechei strain pM206-OXA181 (accession no. AP018831.1). The results revealed that p262-OXA-181 was similar in length and structure to pM206-OXA181 and had the same features previously described about IncX3 plasmids (11, 12). However, on p743-OXA-181, the blast search tool could not identify the replication protein (rep) and the quinolone-resistant gene regions on the plasmid structure; instead, the *qnrB1* was found in the chromosome (Fig. 2A). In addition to the *bla*_OXA-181_, strain EFN262 coharbored *bla*_DHA-1_ on a 22,211-bp genetic structure (accession no. CP092516) whose identity could not be determined by PlasmidFinder. Synteny analysis was performed by comparing the sequences of the genetic element to the sequence of a plasmid (accession no. CP069844.1) recovered from a K. pneumoniae strain from the United States. BLAST analysis showed both sequences shared a 100% query cover and 99.98% identity. *bla*_DHA-1_ was carried on an IS*26* composite transposon and flanked immediately upstream and downstream by transcriptional regulator *LysR* and a hypothetical protein, respectively, and further downstream, a fluoroquinolone-resistance mediating gene *qnrB* (Fig. 2B). *bla*_OXA-48_ was found on a 74,647-bp IncL plasmid, p299-OXA-48 (accession no. CP092591), with a GC content of 50%, in K. pneumoniae. Comparison of p299-OXA-48 sequence with other sequences from the database uncovered a 10.9-kb sequence in p299-OXA-48 absent in the other plasmids (Fig. 3A). *In silico* analysis showed that p299-OXA-48 had a 99.99% sequence identity to other plasmids, with an 85% query cover. *bla*_OXA-48_ was detected flanked by two copies of IS*1999* isoforms IS*10A* upstream and downstream, forming a Tn*1999.2* composite transposon (Fig. 3B). The IS10A was truncated by IS*1* proteins InsA and InsB upstream and the transcriptional regulator *lysR* downstream. The entire plasmid sequence comparison revealed incorporation of unknown hypothetical proteins, and sequences encoding AAA ATPase and polyphosphate kinase, all about 10,851 bp long into p299-OXA-48 (Fig. 3A). Ten IncL plasmids were recovered from the NCBI database, and together with p299-OXA-48 sequence, a maximum-likelihood tree was constructed. The result proved that p299-0XA-48 was distinct from all the other plasmids (Fig. 4).

### Plasmid transfer, fitness cost, competition assay, and plasmid stability.

**FIG 5:**
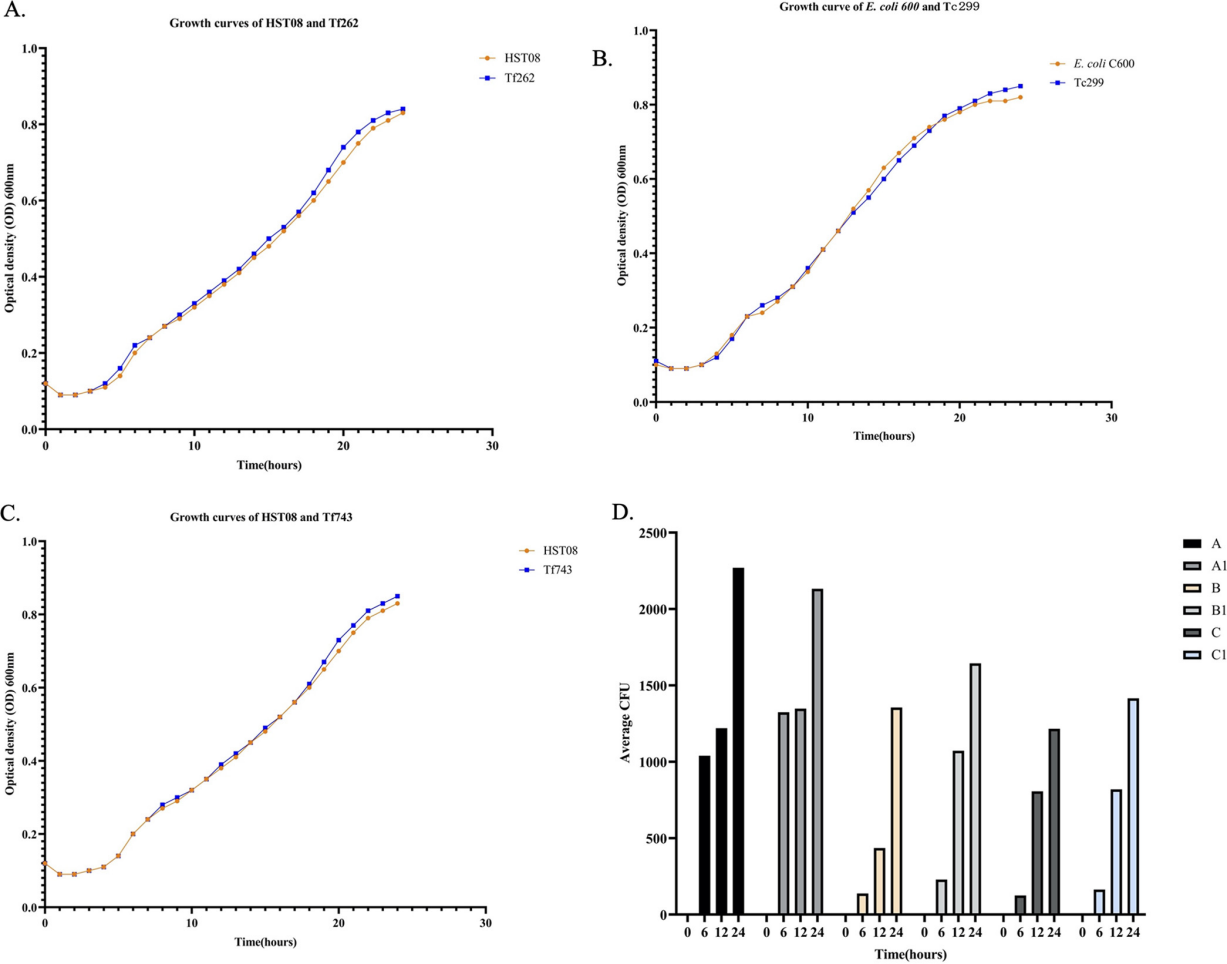
(A) Growth rate of host E. coli HSTO8 (orange line) and transformant Tf262 (blue line) grown in antibiotic-free medium. (B) Growth rate of host E. coli C600 (orange line) and transconjugant (blue line). (C) Growth rate of host E. coli HST08 (orange line) and transformant Tf743 (blue line). (D) *In vitro* competition assay between hosts, transconjugants, and transformants. A: E. coli C600 + Tc299 on ampicillin selective plates. A1: E. coli C600 + Tc299 on LB agar nonselective plates. B: E. coli HST08 + Tf262 on ampicillin selective plates. B1: E. coli HST08 + Tf262 on LB nonselective plates. C: E. coli HST08 + Tf743 on ampicillin selective plates. C1: E. coli HST08 + Tf743 on LB nonselective plates.

To evaluate HGT efficiency of p262-OXA-181, p299-OXA-48, and p743-OXA-181, agar mating and electroporation assays were performed. IncL plasmid (p299-OXA-48) proved to be highly transferable, with a conjugation efficiency of 1.8 × 10^−2^. Presence of several colonies of electro-transformants on LB plates also indicated high transferability of IncX3 plasmids. To assess whether carriage of these plasmids poses any fitness burden on the host, a growth test was conducted. The growth curve intimated that carriage of IncX3-*bla*_OXA-181_ and IncL-*bla*_OXA-48_ did not obtrude any fitness burden on the host cells (Fig. 5A to C). Notably, when transconjugants, transformants, and their respective hosts were cultured in the same growth medium, the transconjugant and transformants outcompeted their hosts. Thus, possession of IncX3-*bla*_OXA-181_ and IncL-*bla*_OXA-48_ conferred a competitive advantage on the host (Fig. 5D).

To confirm how stably these plasmids could be maintained among the bacteria generation, Tc299, Tf262, and TF743 were serially propagated in LB medium. Number of colonies recovered on the antibiotic-supplemented plates suggested a greater proportion of the bacteria maintained their plasmids, with retention rates of 87.6% (Tf262-IncX3-*bla*_OXA-181_), 94.3% (Tc299-IncL-*bla*_OXA-48_), and 95.7% (Tf743-IncX3-*bla*_OXA-181_). Colony PCR results showed the presence of IncL and IncX3 plasmids after 20 passages.

## DISCUSSION

Carbapenem resistance among Enterobacteriaecae constitutes an enormous public health threat due to the high mortality and economic burden it imposes (16). Using whole-genome sequencing, we investigated antimicrobial resistance, horizontal gene transfer, phylogenetic relationships, and genetic context of carbapenem resistance genes *bla*_OXA-48_ and *bla*_OXA-181_ in K. pneumoniae, *K. quasipneumoniae*, and E. cloacae isolates. Particularly, K. pneumoniae strains have been implicated in the majority of carbapenem-resistant Enterobacteriaceae (CRE) infections (6). However, recent studies have also disclosed *K. quasipneumoniae* as an etiologic agent involved in potentially fatal Klebsiella species infections, contrasting with the previous assertion of being only a gut commensal (17). It is pertinent to note also that MALDI-TOF had initially identified EFN262 as K. pneumoniae. This was corrected by the result of the ANI analysis using assembled fasta sequences. Such misidentification of Klebsiella species other than K. pneumoniae, by standard diagnostic and phenotypic methods, as has been previously identified (18–20), could possibly be the reason *K. quasipneumoniae* infections are underreported. All three isolates were multidrug resistant, and although by the CLSI breakpoint they were not resistant to the carbapenems, confirmatory tests showed they were carbapenemase producers, and this result was substantiated by the PCR and sequencing results. K. pneumoniae isolates containing *bla*_OXA-48_ are known to show elevated MIC to imipenem (10). Notably, EFN299 showed an MIC of 2 μg/mL. Of particular interest was the transconjugant strain Tc299, which also exhibited the same MIC 2 μg/mL, which by the CLSI breakpoint classifies both as intermediates. This result supports the call for improved phenotypic procedures for detection of OXA-48-like producing strains and complementation of phenotypic methods with genotypic characterization of these strains that will otherwise remain elusive.

Strain EFN262 was assigned ST5015, EFN299 ST11, and EFN743 ST456. ST5015 is an emerging ST of carbapenem-resistant *K quasipneumoniae* strains. It was recently reported in *K. quasipneumoniae* isolates carrying *bla*_NDM-1_ on plasmids during a survey in a Vietnamese neonatal hospital (15). In an effort to determine whether the same clone from Vietnam is causing infections in Ghana, we generated a phylogeny of the ST5015 from Vietnam and Ghana and also compared their SNPs differences. We found from the results that the strains responsible for the infections are different in both countries. Conversely, ST11 is an international high-risk clone accounting for around 60% of carbapenem-resistant *K pneumoniae*, especially in China (21, 22). Phylogeny with 29 international ST11 genomes showed a close relationship between our strain and others from North and South America. There are also increasing reports of CRE infections involving ST456 Enterobacter species (23), and these all together point up the need to broaden surveillance and monitoring of such pathogenic strains to curb their dissemination.

Plasmids, transposons and insertion sequences are vital mobile genetic elements that promote the rapid dissemination of antibiotic resistance genes by horizontal transfer (24). IncX3 is an epidemic plasmid that has been shown to contribute significantly to the dissemination of *bla*_OXA-181_ (8, 25, 26). The 51.5-kb plasmid has been found to acquire the *bla*_OXA-181_ gene via an IS*Ecp1*-related one-end-sided transposition mechanism (8, 27). *bla*_OXA-181_ was identified on 51.5-kb p262-OXA-181 and 45-kb p743-OXA-181 IncX3 plasmids in this study. Except for a few deletions in p743-OXA-181, there was a high level of homology between plasmid sequences from this study and the sequences from the NCBI database. IncL plasmids harboring *bla*_OXA-48_ genes are also widely distributed, and reports from a number of studies have found *bla*_OXA-48_ on a 63-kb conjugative IncL plasmid embedded in a Tn*1999* composite transposon or its variants, facilitating the dissemination of *bla*_OXA-48_ (7, 28, 29). Here, *bla*_OXA-48_ was located on a 74,647-bp conjugative IncL plasmid with ~10.9 kb sequences encoding hypothetical proteins and AAA ATPases, which were not present on other similar IncL plasmids compared, although the impact of the sequences on the plasmid activity could not be determined, ATPases have been reported to be important catalysts enhancing almost every step in bacterial conjugation (30). Further studies are needed to investigate the role of the other proteins on the plasmid activity, as to whether they are associated with plasmid fitness or stability and/or virulence. DHA-1 forms part of AmpC β-lactamases that confer resistance to penicillin, cephalosporins, cephamycins, and monobactams. The first plasmid-borne *bla*_DHA-1_ was described in a Salmonella enteritidis in Saudi Arabi and has since been identified in other Enterobacteriaceae globally. Among Enterobacteriaceae, inducible AmpC β-lactamase activity is usually chromosomally encoded. Nonetheless, Klebsiella species are reported to not have AmpC on their chromosome but can have resistance genes through plasmids. Plasmid-borne AmpC genes usually lack genetic components required for regulation of AmpC expression, and therefore expression results from continuous transcription of the gene. Hence, as a β-lactamase that is inducible, regulation of its expression involves one of the genes *ampR*, which encodes a transcriptional regulator of the *LysR* family, *ampG*, which encodes a transmembrane permease, and *ampD*, which encodes cytosolic *N*-acetyl-anhydromuramyl-l-alanine amidase (31, 32). This explains the presence of *LysR* immediately upstream of *bla*_DHA-1_, as found in the 22-kb structure.

The IncL plasmid was transferred at a strikingly high rate in the conjugal transfer assay, as has been reported previously (7). IncX3 plasmids here could not be transferred in a conjugation experiment, but by electroporation. The high rate of transfer heralds the potential of the plasmids to expand their hosts targets.

Antimicrobial resistance is accompanied by a fitness cost usually exhibited in a form of decreased virulence, growth rate, and competitiveness, and studies have shown that the cost of resistance is peaky due to several reasons, including genetic mechanism of resistance (33). Previous studies have illustrated that introducing plasmid selection factors offsets plasmid fitness cost by way of compensatory mutation, thereby enhancing plasmid survival (34). However, our findings indicated that in the absence of selection for plasmid (antibiotic-free medium), no fitness burden was imposed on IncX3-*bla*_OXA-181_- and IncL-*bla*_OXA_-_48-_containing host cells. This result is consistent with previous findings (7, 35, 36), but contrasts with others where some IncL plasmid-carrying hosts suffered a fitness cost (7, 37). Such discrepancies have been suggested to be associated with the genetic background of host cells; thus, depending on the host cell, a plasmid can produce different fitness effects, from no fitness cost to severe fitness cost (38). After several passages in the absence of antibiotic selection, plasmids IncX3-*bla*_OXA-181_ and IncL-*bla*_OXA-48_ were stably maintained in a greater proportion of bacteria. Such similar findings have been reported in other studies (7, 8). Despite the significant advantage plasmids provide their hosts, their metabolic activities in the host cells can cast a huge burden on the host. As a result, strains carrying no plasmids are expected to have a competitive edge over plasmid-carrying strains (34). Even so, transconjugants and transformants in this study exhibited higher competitive abilities in the same culture environment than their hosts. Taken together, these traits of the plasmids have a greater implication for their proliferation and contribute largely to the rapid dissemination of resistance genes.

Antimicrobial resistance has become one of the major public health concerns in recent times. Carbapenems are one of the most efficacious and safest treatments for infections by multidrug-resistant bacteria. Therefore, resistance to carbapenems is a serious threat to our treatment options. Genomic surveillance studies have indicated that OXA-48-like producing Enterobacteriaceae are being disseminated regularly, and this brings to the fore the urgent need to institute a robust system to strengthen our surveillance. This study has characterized Enterobacteriaceae containing OXA-48-like carbapenemases and *bla*_DHA-1_ cephalosporinase from a regional hospital in Ghana. The findings in this study suggest that the dissemination of IncX3 and IncL plasmids could be attributed to the efficient lateral transfer, apparently no fitness cost, plasmid stability, and the competitive advantage conferred on host cells.

## MATERIALS AND METHODS

### Ethical considerations.

Written informed consent was obtained from all participants of the study. Protocols for this study were reviewed and approved by the Institutional Review Board of the Noguchi Memorial Institute for Medical Research, University of Ghana (FWA00001824) and the Faculty of Medicine, Tokyo Medical and Dental University (M2017-208).

### Bacterial isolation, identification, and antimicrobial susceptibility testing.

A total of twenty-nine Gram-negative bacterial isolates were cultivated from urine (*n* = 10), high vaginal swab (HVS; *n* = 1), pus (*n* = 1), sputum (*n* = 1), stool (*n* = 1), and unknown (*n* = 15) from the Effia Nkwanta Regional Hospital between August 2018 and September 2019. Initial identification of the isolates was done using the EB-20 ID Test kit (Nissui Pharmaceutical Co., Ltd., Tokyo, Japan) and confirmed by MALDI Biotyper (Bruker Daltonics, Karlsruhe, Germany) and average nucleotide identity (ANI) (https://github.com/ParBLiSS/FastANI) analysis. The MIC of 16 antibiotics were determined by broth microdilution using commercial DP31plates (Eiken Chemical Co., Tokyo, Japan). Carbapenemase production was confirmed following the modified carbapenem inactivation method (mCIM) outlined in the Clinical Laboratory Standards and Institutes guidelines (CLSI, 30th edition). The results were interpreted according to the guidelines of the CLSI, M100-S30. E. coli reference strain ATCC 25922 was used as the quality control strain. K. pneumoniae ATCC 700603 and K. pneumoniae ATCC BAA-2148 were used as negative and positive control strains, respectively, for the mCIM test.

### Carbapenemase genes screening.

DNA was extracted using CICA Geneus DNA Extraction Reagent (Kanto Chemical Co., Tokyo, Japan) as described (11). Isolates were screened for carriage of carbapenemase genes (*bla*_VIM_, *bla*_IMP_, *bla*_KPC_, *bla*_OXA-48-like_, *bla*_GES_, and *bla*_NDM-1_) as described previously (39).

### Whole-genome sequencing, hybrid assembly, and bioinformatic analysis.

Genomic DNA was extracted from carbapenemase-positive strains with the MagAttract HMW DNA kit (Qiagen, Hildon, Germany) according to the manufacturer’s instructions. Paired-end libraries were prepared using the Illumina DNA prep with the IDT for Illumina DNA/RNA UD Indexes (Illumina Inc, USA). Libraries were sequenced on Illumina Miniseq (Illumina Inc., San Diego, USA) generating paired-end reads of length 151 bp.

Quality of the reads were assessed using fastqc v0.11.9 (https://github.com/s-andrews/FastQC). Quality trimming and filtering was done with fastp v0.23.1 (https://github.com/OpenGene/fastp). Long-read sequencing was carried out on MinION with the Flongle flow cells (Oxford Nanopore Technologies, Oxford, United Kingdom). DNA libraries were prepared using the ligation sequencing kit SQK-LSK109. Low-quality reads (MinION Q <10) and long reads (≤1,000 bp) were filtered out with Filtlong (https://github.com/rrwick/Filtlong) after trimming with Porechop v0.2.4. Hybrid *de novo* assembly of long-read and short-read sequences was performed by unicycler v0.4.8 (https://github.com/rrwick/Unicycler). Sequences were annotated with Prokka v1.14.5 (40) and online server RAST (https://rast.nmpdr.org/). Antibiotic resistance gene profiles, sequence types (ST), and replicon types were determined by a search on the ResFinder v4.1 (https://cge.food.dtu.dk/services/ResFinder/), MLST (https://cge.food.dtu.dk/services/MLST/), and PlasmidFinder (https://cge.food.dtu.dk/services/PlasmidFinder/) databases at the Center for Genomic Epidemiology (CGE), respectively. A core-genome-based maximum likelihood phylogeny of 29 international K. pneumoniae ST11 isolates and the K. pneumoniae ST11 from this study was constructed using iqtree v2.0.3 (41). Branch patterns were analyzed with 1,000 bootstrap repeats. A second core-genome-based phylogeny of three *K. quasipneumoniae* ST5015 was generated using core gene alignment generated with Roary v3.11.2 (42). Genomes used were retrieved from GenBank (last accessed February 2022) and the National Center for Biotechnology Information (NCBI). K. pneumoniae genomes used were retrieved from the K. pneumoniae database (https://bigsdb.pasteur.fr/klebsiella/). The genetic context of *bla*_OXA-48_, *bla*_OXA-181_, and *bla*_DHA-1_ and linear plasmid comparisons were generated and visualized using EasyFig v2.2.2 (43). Phylogeny of IncL plasmid from this study and 10 others retrieved from the NCBI database (last accessed February 2022) was generated with megaX v0.1 (44).

### Plasmid transfer experiments.

Conjugal transfer of IncL-*bla*_OXA-181_ was investigated by the agar mating method as described earlier (12). Recipient strain E. coli C600 and donor EFN299 were mated in a 1:1 ratio. Transconjugants were selected on bromothymol blue (BTB) agar supplemented with 50 μg/mL rifampicin, 8 μg/mL ampicillin, and 4 μg/mL sulbactam. Recipient strains were selected on BTB agar supplemented with 50 μg/mL rifampicin. Conjugation efficiency was computed as number of transconjugant colonies per recipient colonies. Conjugation experiments for strains EFN262 and EFN743 were not successful, and therefore plasmid transfer for these strains was investigated by electroporation. For electroporation, plasmid DNA was extracted using the NucleoBond Xtra Midi (Macherey-Nagel, Germany). Briefly, 1 μL of plasmid DNA, extracted from the two isolates, were mixed with 50 μL of electro-competent cell E. coli HST08 (TaKaRa Bio, Shiga, Japan) on ice. The mixtures were transferred into cuvettes on ice and kept on the ice for 3 min, after which they were electroporated using the BIO-RAD MicroPulser Electroporator (Bio-Rad Laboratories Inc., Tokyo, Japan). Electroporation times recorded for both were 3.0 ms and 3.7 ms at 1.8 kV, respectively. Immediately following electroporation, 1,000 μL of super optimal broth was added to the mixtures in the cuvettes and pipetted thoroughly to mix and transferred into microcentrifuge tubes. The tubes were incubated for 1 h at 37°C with shaking at 200 rpm. Subsequently, 100 μL of the suspension was spread on LB agar supplemented with 100 μg/mL of ampicillin. The plates were incubated at 37°C overnight and checked for electro-transformants.

### Bacterial fitness cost assay.

McFarland 0.5 suspensions of transconjugant Tc299, host strain E. coli C600, electro-transformants Tf262 and Tf743, and host E. coli HST08 were prepared in 3 mL LB broth. A volume of 0.4 μL each of the suspensions were diluted in 200 μL of LB broth in a 96-well plate. The plate was loaded into the BioTek Synergy HTX multimode microplate reader (Thermo Fisher Scientific). Growth rates were read by Gen5 v3.11 software by measuring optical density (OD) at 600 nm at 30 min intervals for 24 h. The assays were conducted in triplicate, and the average OD was calculated and fed into GraphPad Prism v9.3.1 to generate growth curves.

### *In vitro* competition assay.

Competition assays were conducted between the transconjugant, transformants, and their host to determine if possession of IncX3-*bla*_OXA-181_ or IncL-*bla*_OXA-48_ confers a competitive advantage on their host. McFarland 0.5 suspensions each of Tc299, E. coli C600, Tf262, Tf743, and E. coli HST08 were prepared in a 0.9% physiological saline. A 100 μL volume each of Tc299 and E. coli C600; Tf262 and HST08; Tf743 and HST08 were mixed in sterile tubes. Twenty microliters (20 μL) of each mixture were diluted in 3 mL LB broth and incubated at 37°C at four different time lengths (0, 6, 12, and 24) hours. After incubation, broth cultures were serially diluted in saline, and 100 μL of the dilutions (10^−5^) was spread on both selective and non-selective LB agar plates and incubated at 37°C for 18 h. Tc299, Tf262, and Tf743 were selected for, on 8 μg/mL ampicillin-supplemented LB plates. The assays were conducted in duplicate, and the average colony count was calculated and plotted as bar graphs.

### Plasmid stability assay.

To assess the stability of the plasmid in a bacterial population, a single colony of the transconjugant strain Tc299, transformants Tf262 and Tf743 were propagated in 1 mL antibiotic-free LB medium at 37°C while shaking at 200 rpm. Hundred-fold (100-fold) dilutions of the previous cultures were prepared in a fresh 1-mL LB medium until the 20th passage. Cultures from the last passages were serially diluted in saline, and 50 μL of the dilutions (10^−5^) was spread on both antibiotic-containing (8 μg/mL ampicillin) and antibiotic-free LB agar plates. Assays were conducted in duplicate. Plasmid retention rate was estimated by dividing the average number of colonies on antibiotic-containing plates by the average number on the antibiotic-free plates. Presence of plasmids after passaging was confirmed by colony PCR targeting the *taxC* gene of the IncX3 plasmid and the *repA* gene of IncL plasmid, as described previously (45, 46).

### Data availability.

Data sets generated from the study are available from the corresponding authors upon reasonable request.

Genomes of strains EFN262, EFN299, and EFN743 have been deposited in GenBank under Bioproject PRJNA473419. Accession numbers of all strains used in this study are listed in Data Set S2 in the supplemental material.
